# “This Program has Given Me the Proper Nutrition that I Deserve”: Participant Perspective on a Medically Tailored Meal Intervention

**DOI:** 10.1177/21501319261465077

**Published:** 2026-06-25

**Authors:** Patricia Knoepp, Salma Ali, Myklynn LaPoint, Jacqueline Nguyen, Colleen Dagley, Jean Terranova, Seth A. Berkowitz

**Affiliations:** 1 Sheps Center for Health Services Research, University of North Carolina at Chapel Hill, Chapel Hill, NC, USA; 2 699833 Community Servings, Inc, Jamaica Plain, MA, USA; 3 Division of General Medicine and Clinical Epidemiology, University of North Carolina School of Medicine, Chapel Hill, NC, USA

**Keywords:** community health, disease management, health inequities, social determinants of health, underserved communities, food is medicine, medically tailored meals

## Abstract

**Objective:**

Diet-related disease is a leading cause of morbidity and mortality in the U.S. Medically tailored meals are one intervention that can address diet-related disease. We sought to help inform two key aspects of medically tailored meal program design: 1) should the meals be provided for a focal individual alone, or for the entire household? And 2) does a dedicated delivery driver promote reduced loneliness compared with using a commercial shipper?

**Methods:**

Qualitative analysis of semi-structured interviews of purposively selected participants in the Food as Medicine for Families (FAME-F) randomized clinical trial. 25 interviews were conducted between May 2024 - January 2025. We conducted a thematic analysis to analyze the data. Transcripts were coded by three independent coders who met to compare and reconcile any coding discrepancies. Code reports were generated for each code and narrative summaries were written.

**Results:**

Participants were overall enthusiastic about the intervention, and they felt the intervention improved multiple aspects of health. Food preferences were an important determinant of satisfaction with the meals. Participants did not clearly see benefits to the ‘feed the family’ approach, noting that because medically tailored meals are tailored to a specific individual’s needs, they may not accord with other household members’ preferences, and that the household budget space freed up by providing meals can allow other household members to purchase food they prefer. Participants also did not perceive clear benefits for the ‘dedicated driver’ approach, noting little interaction with the driver.

**Conclusions:**

Medically tailored meals can help people improve health by overcoming barriers to healthy eating. Results provide reassurance that providing medically tailored meals for a focal individual can lead to meaningful dietary improvement in spite of potential for under-dosing if meals are shared, and that delivery approach can be selected based on logistical considerations.

## Introduction

Diet-related disease, including type 2 diabetes mellitus, coronary heart disease, congestive heart failure, hypertension, and many other clinical conditions, are leading causes of morbidity and mortality in the U.S.^1-3^Diet-related disease results from barriers to healthy eating, including food insecurity, unhealthful food environments, and lack of time and capacity to prepare healthful meals.^4,5^ Food is Medicine interventions seek to prevent and/or treat diet-related disease through the provision of nutrition resources.^6,7^ One type of Food is Medicine intervention, medically tailored meals, has received growing attention.^1^ Medically tailored meals (MTM) entail the home delivery of ready-to-consume meals, prepared under the supervision of a registered dietitian nutritionist to be appropriate for the combination of comorbidities an individual faces.^1^ Studies have found that medically tailored meals improve diet quality and health outcomes, and are associated with reductions in acute healthcare utilization, such as emergency department visits, and healthcare spending.^7-12^ This has led to growing interest in expanding medically tailored meals as clinical management interventions.

With their promise for improving health, understanding how best to design MTM interventions is an important question. However, there is a lack of data on two key implementation questions for MTM programs. The first question is one of dose—should the meals be targeted to a focal individual alone, or for the entire household? Because food is often shared in households, providing meals for only a focal individual could result in ‘underdosing’ the nutrition resources provided. However, providing meals to additional household members increases program cost, and because MTM are tailored to a specific individual’s needs, they may not accord with other household members’ preferences. Further, because MTM represents a substantial proportion of the focal individual’s food needs, household budget space can be ‘freed up’, allowing more to be spent on other members’ food.^9,13^

The second question relates to delivery. Many organizations providing MTM employ delivery drivers who regularly visit a particular individual. While not as intensive a connection as a daily home visiting program, such as Meals on Wheels,^14-16^ a weekly connection with a friendly face may still be a beneficial social connection that could reduce loneliness and improve mental health. On the other hand, advances in commercial logistics mean that MTM can now be delivered cost effectively by commercial logistics firms. This can enable organizations to expand their reach into areas they could not previously serve, but potentially with the downside of reducing this social connection.

The Food as Medicine for Families (FAME-F) randomized clinical trial was conducted to answer these questions. FAME-F was planned as a mixed methods study. A prior manuscript has reported quantitative results,^17^ and this manuscript presents qualitative findings.

## Methods

### The Food as Medicine for Families Randomized Clinical Trial

Food as Medicine for Families (FAME- F) has been described elsewhere,^18^ but it was a pilot 2x2 factorial randomized comparative effectiveness trial, providing 10 MTM per week to participants for 12 weeks. Random allocation in the trial occurred along two dimensions: dose (‘feed the family’ vs. ‘feed the individual’), and delivery (‘dedicated driver’ vs. ‘commercial shipper’). The ‘feed the family’ condition provided 10 meals per week for each individual in the household, while the ‘feed the individual’ condition provided 10 meals per week for the focal individual only. In the ‘dedicated driver’ condition, participants received their weekly meals from driver who was employed by the v agency, while in the ‘commercial shipper’ condition participants received meals from the same MTM organization, but delivered by a commercial logistics organization. The primary quantitative outcome for the dose dimension was diet quality,^19^ and the primary quantitative outcome for the delivery dimension was loneliness.^20^ Participants were adults (aged ≥ 18 years) recruited from the referral base of Community Servings, a not-for-profit community-based MTM provider in New England. The study was prospectively registered on clinicaltrials.gov as NCT06160973 on November 29, 2023. The institutional review board at the University of North Carolina at Chapel Hill approved this study (IRB# 23-2420), and 93 individuals were randomized in the trial.^17^ Participants gave verbal informed consent, recorded electronically, before participating in the overall trial, and gave separate verbal informed consent, recorded electronically, to participate in the qualitative interviews.

The prevalence of food insecurity in the study sample at baseline was 71%, assessed using the 18-item USDA household food security survey module, with 30 day lookback period.^17,21^

In brief, the quantitative results of FAME-F were that there was no meaningful difference between the ‘feed the family’ and the ‘feed the individual’ condition with regard to diet quality as measured by the Healthy Eating Index and Dietary Screener Questionnaire based measures of food consumption,^19,22^ and no meaningful difference between the ‘commercial shipper’ and ‘dedicated driver’ condition with regard to loneliness as measured by the De Jong Gierveld loneliness scale.^20,23,24^

### Data Collection and Study Sample

For this qualitative study, FAME-F trial participants were recruited to complete an interview by a study research assistant. Sampling was purposive, with a goal of enrolling participants from all treatment conditions and with a range of demographic characteristics. Interviewers with bachelor’s or masters degree training in public health and biomedical research, with no prior relationship to the participants, contacted a participant up to three times to complete the interview. Interviewers explained the purpose of the interviews to participants. Open-ended, in-depth questions, using a semi-structured interview guide, were posed during the interviews (eAppendix A). A total of 25 interviews were conducted via telephone between May 2024 - January 2025. Investigators determined that thematic saturation was reached at this point (see more details below), and the demographic characteristics of interview participants was broadly similar to that of trial participants overall. The interviews lasted approximately 30 minutes. Participants were offered a $20 gift card for the interview. Transcripts were not returned to participants.

The COREQ checklist was used for study reporting.^25^

### Data Analysis

All interviews were audio-recorded with participant permission and transcribed verbatim. Identifiable information was removed from the transcripts prior to analysis. Transcripts were reviewed with the audio files for accuracy and completeness, and any personal identifying information was removed. Once completed, all transcripts were imported into ATLAS.ti 23.2.1. We conducted a thematic analysis to analyze the data, primarily focusing on interpretation of participant statements as they relate to specific themes.^26,27^ We approached the concept of thematic saturation as a phase during data collection in which no new ideas, images, or themes were emerging. In this study, that occurred after 25 interviews.^28^

Prior to analysis, a codebook was created based on specific topics related to the interview guide (eAppendix B). During the coding process, inductively derived codes were developed as needed to fully capture all relevant information (eAppendix C). The transcripts were coded by three independent coders who met to compare and reconcile any coding discrepancies. Once coding was complete, code reports were generated for each code and narrative summaries were written. These code summaries included a narrative description of the themes that emerged related to each code. Illustrative quotes were used to highlight each theme.

## Results

### Participants

Twenty-five participants completed interviews; all were included in analysis (Table 1). Of these participants, 72% were women; 60% identified their race as White, 20% as Black, 4% as Asian, and 16% as “other” or unknown; and 12% identified their ethnicity as Latino or Hispanic. Participants’ ages ranged from 33 to 78 years (mean: 56 years, SD: 11 years). Participants were evenly dispersed across possible treatment allocations: delivered household (24%), delivered individual (28%), shipped household (24%), and shipped individual (24%).

**Table 1.** Demographics Characteristics (n=25)

Characteristic	N or mean	% or SD
Age (Range: 33—78 years)	56	11
Race		
White	15	60%
Black	5	20%
Other	4	16%
Asian	1	4%
Ethnicity		
Hispanic or Latino	3	12%
Non-Hispanic or Latino	22	88%
Gender		
Woman	18	72%
Man	7	28%
Intervention Dose		
Delivered Household	6	24%
Delivered Individual	7	28%
Shipped Household	6	24%
Shipped Individual	6	24%

### Findings

Study analysis findings are broken apart into the following categories: overall participant experience with the intervention, findings related to the ‘dose’ dimension, findings related to the ‘delivery method’ dimension, and impacts of the intervention (health and economic).

#### Participant Experience

Participants were asked to reflect on their overall experience in the study. Interview questions specifically asked about the following: experience receiving meals, amount of food consumed, meal satisfaction, and amount of food. Selections of these findings are presented below, with more detail, including themes and illustrative quotes, provided in eTable 1.

Participants voiced different feelings about the flavors of food and variety based on their personal preferences. Overall, participants liked having easy access to healthy food. When asked about the percentage of food they actually consumed, 82% of participants reported eating all or a majority of the meals provided**.** Participants reported that receiving preferred foods, convenience, and ease of access positively impacted meal consumption. “*As soon as the meals started coming, we started eating them immediately. My back is in so much pain to be able to cook anymore. I can't do it. So, it's either pre-made food, processed or this program that has saved me.”*

Participant satisfaction with meals provided varied. Most participants voiced appreciation about receiving the meals and expressed mixed feelings about the meals themselves. More specifically, they reported personal preference and meal variety as the primary factors in meal satisfaction. “*No. Just me and my taste. I wasn't used to eating that kind of food, eating so healthy all the time. I did like vegetables. I did like salad and all that, but I didn't eat it every day. So getting used to eating without so many ingredients like we do.”*

Some participants recommended that participants be given opportunities to choose items that would be included in their meals each week.

#### Dose Dimension: ‘Feed the Family’ vs. ‘Feed the Individual’

Participants were randomized to receive their meals either for a focal individual alone or for everyone in their household. Participants in the “household” arm of the study were asked about how they felt about meals being provided to members of their household. They reported challenges with food options, particularly with medically tailored foods not necessarily being favored by others in the household (eTable 2). “*There were some things that they didn't like, some of the same things I didn't like. My daughter wasn't a big fan of some of the breakfasts, but other than that, she was pretty good with everything else….Maybe for the children, I guess you can say in simpler ways, more kid-friendly breakfasts… the breakfast weren't so kid-friendly for the kids….I want to say there was a quiche. I mean, it's not like so-- at least for my daughter, it wasn't so kid-friendly.”*

Participants in the ‘individual’ study arm did not report any issues or difficulties with members in their household eating different foods from their MTMs. One participant in the ‘individual’ study arm did note feeling meals for the household would not be beneficial for their circumstances due to dietary preferences and differing routines. “*No, they wouldn't have eaten it… And they're very particular. My son is all grass-fed, all natural. And my daughter, I don't know. She's a flight attendant so she's never around, so…. Yeah. Pickiness and, yeah, whatever. Me, I'm not all that picky.”*

#### Delivery Dimension: ‘Commercial Shipper” vs. “Dedicated Driver”

Participants from both groups mentioned “looking forward to deliveries”, that they “didn’t interact much with delivery driver” and that the “delivery driver was nice and considerate”. Participants assigned to the ‘dedicated driver’ group were asked if they had formed a relationship with their delivery driver, 3 people simply said “yes” and 3 people simply said “no.” Participants reported some delivery and shipping issues that impacted their program experience (eTable 3). This included issues with when foods were delivered and communications about delivery. “*It was pretty good. The only thing I would say is my meals were delivered via UPS. And with the weather, sometimes it was left outside, and I didn’t know that they had came. So sometimes, it may have been out there for several hours. So some of the stuff had started-- the ice started melting, or if it was frozen, it was kind of melting.”*

Participants were asked whether their feelings of connection to their community changed while receiving meals. These conversations centered around feelings of loneliness and the impact of the different delivery methods on feelings of connection. Overall, there was a range of feelings related to this, without being clearly different by the method of delivery a specific participant received. Similarly, delivery method did not seem to impact any reports of feelings of connection, social contact, or loneliness.

#### Impacts of Intervention

Participants were asked whether they thought receiving the meals made a difference to their (or their household members’) health (eTable 4). They reported improved dietary habits, improved hemoglobin A1c/blood sugar levels, improved blood pressure, weight loss, and positive emotional and psychological impacts. “*Yeah, yeah. It did. It did. Definitely…Lower blood pressure, losing some belly, having more energy. The fact that the food has a lot of protein, it is a very huge deal in my house…And I have discussed all of this wellness with my doctor. And she feels like, yeah, my health is way better now.”* “*Oh, yeah, I like them [the* MTM*], and I wish they didn't end because it made things simple for me and made me have good meals every day. And it actually kept me healthier with my diabetes and my COPD and everything that I have issues go on. So kind of liked it.”*

Participants were asked if being in the study and receiving the meals made it easier to make ends meet or generally improve their ability to afford other day-to-day needs. When asked if being a part of the study impacted the participants ability to make ends meet, 10 participants responded with a simple “yes”. Additionally, some reported that it helped general finances and increased food stability. “*I definitely think it gave me a lot of peace of mind with getting meals just because in some weeks it was harder financially to buy food during having the medical issues. So at least having the food coming in was just a big help in itself. I think it did help medically, I would say, too, just because they were, I'd say, well-balanced and providing good nutrition without kind of having the bad numbers go up.”*

## Discussion

In this qualitative evaluation of the experience of participants in a randomized trial that provides MTM, participants were overall enthusiastic about the intervention. Participants reported positive impacts on health, although satisfaction with the meals varied by taste preferences. Participants did not clearly see benefits to the ‘feed the family’ approach, nor did they perceive clear benefits for the ‘dedicated driver’ approach.

The findings of this study are consistent with and expand those of prior MTM studies. Most notably, these results are consistent with the quantitative results of the FAME-F study, which showed little difference in diet quality between the ‘feed the family’ and ‘feed the individual’ arm, and little difference in mental health outcomes between the ‘dedicated driver’ and ‘commercial shipper’ arms. Further, these results are consistent with prior qualitative work on MTM interventions.^9,29-31^ In particular, these results lend further support for the idea that MTM improves health through overcoming barriers to healthy eating, and that MTM interventions are feasible and acceptable for participants.^1^ Overall, this supports the testing of MTM interventions in larger-scale trials.^7^

The findings of this study have important implications. One particularly important implication relates to whether to provide meals for the focal individual or all members of the household. For instance, there are ongoing implementations of medically tailored meal interventions that provide meals for children and other dependents in the household, along with a focal individual.^32^ Arguments in support of feeding all household members typically focus in two places—first the impact on the focal individual, and second, the impact on others in the household. Regarding the focal individual, the argument is that, because food is shared in households, the focal individual would share meals with other household members, resulting in ‘underdosing’ of the intervention. Regarding others in the household, the argument is that since barriers to healthy eating like food insecurity are shared, others in the household may also benefit from an MTM intervention that overcomes those barriers. This study suggests that, at least for MTM, providing meals for the focal individual alone does not seem to lead to ‘underdosing’. This is likely because sharing is less common than anticipated, owing to the medical tailoring of the meals which makes them less suitable for other household members. We note that this may differ for other food is medicine interventions, such as food subsidies or provision of unprepared grocery items. Another reason is that by providing a substantial proportion of the focal individual’s food needs, household budget space is freed to support the household budget more broadly, so all household members may still benefit indirectly. Regarding the potential impacts of MTM on other household members, it may make more sense to address this by enrolling other household members separately in an MTM intervention, rather than providing meals for the entire household. This would allow tailoring for the specific individual, and is more in-keeping with the intention of MTM as a specific clinical management program, rather than a population-level nutrition intervention such as SNAP (the Supplemental Nutrition Assistance Program). Although not examined in this study (which sought to pilot specific MTM design features in preparation for larger-scale interventions), incorporating strategies for sustainable healthy eating after the end of the intervention is an important part of intervention design. Another related issue that could be explored in more detail in future studies is coordination among multiple nutrition interventions that different household members may be enrolled in, and what the impacts of combining more than one nutrition intervention within a household may be.

Regarding the delivery approach (that is, whether to use a dedicated driver or a commercial shipper), it is notable that, at least in the sample interviewed, there were not clear differences between the studied approaches from the participant’s perspective. Therefore, the decision of which to use might be made on the basis of other considerations, such as cost, feasibility, reach, or convenience. It is also important to note that the experience of different delivery approaches could vary based on whether an individual lives alone or with others. We do reiterate however that this study did not evaluate a home visiting program. Such programs, which provide meals and structured daily in-home visits, are more intensive forms of social connection intervention than studied here. The benefits of those programs have been well established in prior work.^14-16^

The results of this study should be understood in light of several limitations. This was a small study and while saturation was reached in our sample, there may be other experiences with MTM interventions not captured here. Demographically, the sample was largely White women. Perspectives may vary across gender, ethnic, and cultural groups. Another limitation is that this was a short-term study, and experiences could vary with longer participation. However, these limitations are balanced by key strengths—in particular triangulation with quantitative findings.

## Conclusion

MTM are promising interventions that can help people improve health by overcoming barriers to healthy eating, The results of this help inform important questions in MTM intervention design. In particular, the results provide reassurance that providing MTM for a focal individual alone can still lead to meaningful dietary improvements, and that using whichever delivery approach is best suited, from a logistical standpoint, is unlikely to have major implications for participant experience.

## Supplemental Material

Supplemental material - This Program has Given Me the Proper Nutrition that I Deserve”: Participant Perspective on a Medically Tailored Meal Intervention Supplemental material for This Program has Given Me the Proper Nutrition that I Deserve”: Participant Perspective on a Medically Tailored Meal Intervention by Patricia Knoepp, Salma Ali, Myklynn LaPoint, Jacqueline Nguyen, Colleen Dagley, Jean Terranova, JD, Seth A. Berkowitz in Journal of Primary Care & Community Health
