# Supplementary material for: “This Program has Given Me the Proper Nutrition that I Deserve”: Participant Perspective on a Medically Tailored Meal Intervention
Source: J Prim Care Community Health. 2026 Jun 25;17:21501319261465077. doi: 10.1177/21501319261465077 (PMC13305271; doi:10.1177/21501319261465077)
Supplement: Supplemental material - This Program has Given Me the Proper Nutrition that I Deserve”: Participant Perspective on a Medically Tailored Meal Intervention [file sj-pdf-1-jpc-10.1177_21501319261465077.pdf]

eAppendix for "This Program has Given me the Proper Nutrition that I Deserve": Participant Perspective on a Medically Tailored Meal Intervention

eAppendix A: Interview Guides

Feed the Household Interview Guide

Hi [PATIENT NAME]. Thank you for taking the time to meet with me today.

I'm [YOUR NAME]. I am part of the research team that is looking into ways to help people improve their health using medically-tailored meals. We want to hear about your experience in the study. The information that you share with us will help us improve this program and may help others in the future.

We would love to hear as much about your experience in as much detail as possible. Please feel free to stop me and ask any questions throughout the interview. Remember that there are no right or wrong answers. You don't have to answer any questions that you're uncomfortable with. The interview should take about 30 to 60 minutes to complete.

I'll be doing the interview with you. We will record the interview because we want to make sure that we remember your important opinions and comments exactly as you said them. Everything that you say will remain confidential. Any reports or publications that use data from this interview will not use your name or other identifying information. In order to help protect your privacy and the privacy of others, we ask that you not mention specific names or dates during our conversation.

Do you have any questions at this point?

Are you OK with our recording the interview (yes/no)?

[If no, end interview and thank participant for their time]

*[If yes, **START RECORDING**]*

- **Record patient's approval as follows:**
  - Turn on recorder and state participant's study ID number
  - Ask: *"With a 'yes' or 'no,' can you verbally confirm that you have consented to be interviewed and to have our conversation recorded?"*

GENERAL QUESTIONS

As I said in the introduction, I'd like to hear more about your experience with the study and how you felt about being in the study.

Could you tell me about your experience getting the meals?

Probe: What did you like about the food?

Are there things you would change?

Amounts of certain item (i.e. less soup and more fish, etc.)

Could you tell me about things you think we should change about the program?

Did you think receiving the meals made a difference to your health? Why or why not?
Probe: For example, having lower blood pressure or blood sugars?

Could you tell me about any benefits from getting the meals, aside from health?
Probe: For example, being able to afford medications?

Thinking about the meals, in an average week, what percentage of the food that was delivered do you think you wound up eating? Was there something that made some meals eaten more than others?

[If high] Sounds like you ate a lot of the meals. What contributed to that number?

[If low] Sounds like the meals didn't always work out for you. Why do you think that was?

Probe: Are there things we could do in the future so that people like you might want to eat more of them?

Thinking about the amount of food provided, did the amount seem right to you?
Probe: Too much/too little/just right

[If not just right]: What do you think the ideal number of meals per week would be?

Do you think you would ever want the number of meals each week to vary (more in some weeks than others), or should they stay the same? If they could vary, how might you want that to work?

MEALS AND THE HOUSEHOLD

The program provided meals for others in your household, along with you. How did you feel about that?
Probe: Was that helpful? Why or why not?

What opinions did your family member have about the meals?
Probe: What did they like?
Probe: Are there things they would change?

Did others in the house run into any difficulties with the meals?
Probe: Please tell me more about those difficulties or challenges.

Could you tell me about any benefits others in your household may have gotten from the meals?

Do you think the other people in your household liked the meals?

[If yes]: What did they like about them? Could anything have made them like the meals even more?

[If no]; What didn't they like about them? What could have made them better?

Thinking about the other people in your household, in an average week, what percentage of the food that was delivered do you think you wound up eating? Was there something that made some meals eaten more than others?

In this program, you received enough meals for you and others in your household. But sometimes people don't like to eat the same thing. Think about another program, where you still got meals, but others in your household got groceries they could prepare as they liked, or a subsidy to buy healthy food. Could you tell us your thoughts about a program like that?

Probe: Would a program like that have been better for you and your household?

COMMUNITY CONNECTION

We know that feeling connected to a community is an important part of life for many, but sometimes people can feel lonely or isolated. Did receiving the meals impact how connected you felt to other people?

Probe: for example, did you look forward to the delivery driver dropping off your meals?
If yes, how? If not, why?

Do you think you formed a bond with the people who delivered the meals?

Did receiving the meals ever make you feel less lonely?
If yes, how so?
If not, why?

Did receiving the meals increase your social contact? For example, do you think you talked to more people while receiving the meals?
If yes, how so?
If not, why?

WRAPPING UP

Is there anything else you think I should know about the experience of participating in the study?

That's all the questions I have for now. If we have more questions, could we reach out to you again? Thank you very much for the time you've taken to speak with me. [turn off recorder and end interview]

Feed the Individual Interview Guide

Hi [PATIENT NAME]. Thank you for taking the time to meet with me today.

I'm [YOUR NAME]. I am part of the research team that is looking into ways to help people improve their health using medically-tailored meals. We want to hear about your experience in the study. The information that you share with us will help us improve this program and may help others in the future.

We would love to hear as much about your experience in as much detail as possible. Please feel free to stop me and ask any questions throughout the interview. Remember that there are no right or wrong answers. You don't have to answer any questions that you're uncomfortable with. The interview should take about 30 to 60 minutes to complete.

I'll be doing the interview with you. We will record the interview because we want to make sure that we remember your important opinions and comments exactly as you said them. Everything that you say will remain confidential. Any reports or publications that use data from this interview will not use your name or other identifying information. In order to help protect your privacy and the privacy of others, we ask that you not mention specific names or dates during our conversation.

Do you have any questions at this point?

Are you OK with our recording the interview (yes/no)?

[If no, end interview and thank participant for their time]

*[If yes, **START RECORDING**]*

- **Record patient's approval as follows:**
  - Turn on recorder and state participant's study ID number
  - Ask: *"With a 'yes' or 'no,' can you verbally confirm that you have consented to be interviewed and to have our conversation recorded?"*

GENERAL QUESTIONS

As I said in the introduction, I'd like to hear more about your experience with the study and how you felt about being in the study.

Could you tell me about your experience getting the meals?

Probe: What did you like about the food?

Are there things you would change?

Could you tell me about things you think we should change about the program?

Did you think receiving the meals made a difference to your health? Why or why not?

Probe: For example, having lower blood pressure or blood sugars?

Could you tell me about any benefits from getting the meals, aside from health?

Probe: For example, being able to afford medications?

Thinking about the meals, in an average week, what percentage of the food that was delivered do you think you wound up eating? Was there something that made some meals eaten more than others?

[If high] Sounds like you ate a lot of the meals. What contributed to that number?

[If low] Sounds like the meals didn't always work out for you. Why do you think that was?

Probe: Are there things we could do in the future so that people like you might want to eat more of them?

Thinking about the amount of food provided, did the amount seem right to you?

Probe: Too much/too little/just right

[If not just right]: What do you think the ideal number of meals per week would be?

Do you think you would ever want the number of meals each week to vary (more in some weeks than others), or should they stay the same? If they could vary, how might you want that to work?

MEALS AND THE HOUSEHOLD

In this program, you received meals tailored to your medical needs. Do you think getting this food made it easier for you and your household to make ends meet?

Probe: For example, did not needing to buy food make it easier to pay for medications, or other things like utilities or transportation, or give you more time to manage medications?

Did having some meals taken care of for you make it easier for anyone else to have healthy food? Why or Why not?

Did you run into any difficulties with you eating the provided meals and others in the household eating different food?

Probe: Please tell me more about those difficulties or challenges.

If the program had provided not just meals for you but enough of the meals for others in your household, would that have been better?

What are some things you might like about a program like that? What are some things you might not like?

[COMMUNITY CONNECTION, ASK FOR EVERYONE]

We know that feeling connected to a community is an important part of life for many, but sometimes people can feel lonely or isolated. Did receiving the meals impact how connected you felt to other people?

Probe: for example, did you look forward to the delivery driver dropping off your meals?

If yes, how? If not, why?

Do you think you formed a bond with the people who delivered the meals?

Did receiving the meals ever make you feel less lonely?

If yes, how so?

If not, why?

Did receiving the meals increase your social contact? For example, do you think you talked to more people while receiving the meals?

If yes, how so?

If not, why?

WRAPPING UP

Is there anything else you think I should know about the experience of participating in the study?

That's all the questions I have for now. If we have more questions, could we reach out to you again? Thank you very much for the time you've taken to speak with me. [turn off recorder and end interview]

eAppendix B: Codebook

Code	Code Description
**Amount of food and variation**	Any comments about if the amount of food received felt appropriate AND thoughts on if there should be variation in amount received week to week
**Complaints or Challenges**	Any comments about things that were difficult/challenging OR general complaints about the intervention
**Compliments**	Any general compliments or praise of the intervention
**Delivery vs Shipped Impact**	Any comments relating to experience with the Community Servings or commercial shipper (i.e., bonds made, quality of delivery, etc)
**Differences to Health**	Any comments about whether participants feel the MTMs made any difference to their health
**Easier Access to Healthy Food for Others**	Any comments about increasing access to food for others (could be related to others in the household for folks who only received food for the individual or also related to other friends or community members)
**Experience Receiving Meals**	Any overall or general comments about participants' experience receiving the MTMs
**Household feelings about meals**	Any comments relating to how household members felt about the meals (household arm)
**If food was provided to household Impact**	Any comments relating to how participants would feel about a program that provided meals to the entire household (individual arm)
**Making ends meet/Financial**	Any comments relating to potential financial impacts the program had on participants
**Meal Satisfaction**	Any comments about individual or household members' meal satisfaction (or lack thereof)
**Non-Health Related Benefits**	Any comments around benefits related to receiving the meals that are not related to health
**Percentage of Meals Eaten and Why**	Any comments about the % of meals eaten and why (for household, this includes %s all members of the household)
**Quotables**	Any particularly well-articulated comments regarding experience with or impacts of the intervention
**Recommended Changes**	Any recommendations or comments relating to changes that participants feel would improve the intervention/program
**Socialization Impacts**	Any comments about changes to loneliness, social contact, or social connection

eAppendix C: Code Counts

Code	Count
% of meals and why	38
Amount of food and variation	48
Complaints or challenges	30
Compliments	21
Delivery vs Shipped impact	38
Differences to health	36
Easier access to healthy food for others	8
Experience receiving meals	32
Household- Feelings about meals for household	29
Individual- impact if food was provided to household	12
Making ends meet/financial	19
Meal satisfaction	40
Non-health related benefits	32
Recommended changes	57
Socialization impacts	48

eTable 1: Themes and Illustrative Quotes Relating to Overall Experience with Medically Tailored Meals

Program Experience	
Theme	Illustrative Quote
Experience receiving meals	
Access to food	*Let's see. Where do I begin? Well, the food was better than I expected. I had to add a few seasons to a few things, of course, because you're used to your own taste. But overall, I really enjoyed them. And again, it has saved a lot of money being with medical-- autoimmune and compromise. Too early for that to say that word. I've been able to say what little food stamps I get for fresh fruits and healthier foods. Very sad to see it end.*<br>*I liked the different variations. I would say that with some of the meals, there's no seasoning at all. I know no salt, but none. I had to dress it up each time I had something. But as far as the different meats and everything, they were pretty good. They were pretty good.*
Mixed satisfaction due to varied food preferences	*It was good, but I didn't really like some of the food that was being delivered…Well, I didn't know what it was, and so I just didn't like it…I would try it, but I didn't like it, so I had to avoid it…I don't know. Just--..It was food that I'd never heard of before. I don't even know. I'm not familiar with the food that [inaudible]…[inaudible] just regular meals like--..More chicken and beef [and more?]*
Delivery/shipping issues	*It was pretty good. The only thing I would say is my meals were delivered via UPS. And with the weather, sometimes it was left outside, and I didn't know that they had came. So sometimes, it may have been out there for several hours. So some of the stuff had started-- the ice started melting, or if it was frozen, it was kind of melting.*
Factors impacting consumption	
Food preferences and tastes	*We eat most of the soups and stuff like that. Just some of them I didn't care for. But most of the food was eating the vegetables. And sometimes we had [inaudible] to give it a*

	better flavor. [The pepper?] were plain.
	There was a couple that I didn't really care for. I don't know if they were-- they were kind of weird. I don't know if they were vegan, I think, maybe. And I didn't care for them…Like I said, everything was pretty good except for maybe just the vegan stuff.
Meal satisfaction	
Cultural food differences/ used to different types of food	*No. Just me and my taste. I wasn't used to eating that kind of food, eating so healthy all the time. I did like vegetables. I did like salad and all that, but I didn't eat it every day. So getting used to eating without so many ingredients like we do. I'm Dominican. We have ingredients that are also on the supermarket that are not the best. And we like food that smells four or six blocks away when you are cooking. So eating that kind of stuff without so many ingredients and more natural ingredients. That was kind of tough at first, but I knew that was the best thing to get used to. And it took me probably two months to get used to it. At first, I was like, "Oh, my God. This food is not good." When you taste it, it tastes good, but it doesn't smell like my food. That's it.*
Foods that were disliked or just not eaten	*Some of the meals are fine. Some of them aren't so good. It all depends. Some of them, we try to make it too fancy and I really don't enjoy some of them, especially the soups.*
Amount of food and variation	
Too much food	*Yeah. Some of it was like, "Wow, that's an awful lot in there." But you know what? And some of it, like the turkey chili, there was a lot with the cornbread, and sometimes I wouldn't eat it all. I would just save the other half for lunch the next day. Maybe half of lunch and half for dinner, whatever the case may be.*
Not enough food	*Yep. Well, yeah, but sometimes it can be a little bit too low….But at some point, I felt like the portion size was really, really small. So sometimes if I ate that, I would still be hungry later on. And if I would have ran out*

	of my food, then I would just run out.
	Well, for me, I'm looking for getting my health better and losing weight. For me, it's fine. But I assume that for the general population, people who don't need to lose weight, yes, it's not enough. Maybe a little bit more. And more whole protein.
The right amount of food	*Yeah. It's rather interesting because when I eat and I say I eat just the lunch, it's very satisfying.*
Would like to vary the amount of food	*All my family's going to be cooking. Can you send me five meals or three meals? I like to be able to make that choice.*
Would not like to vary amount of food	*I'd probably stay the same because consistency is the key.*
Recommendations for amount of food and variation	*Yeah. So if I was like one week, I could select the meals that I could get for the following week, so that way I kind of had an idea of different meals that were coming in, or just have a few different options.*<br>*The only thing that I would change is if there was some kind of menu option to be able to select different types of meals ourselves instead of just kind of randomly getting what we would get sometimes. But I mean, the meals themselves, I'd say they tasted pretty good. Myself, personally, the only ones that I didn't really care for too much were the fish ones. But outside of that, I mean, the rest of them were really good.*
Complaints and Challenges	
Do not like or want certain foods	*The only thing I didn't like is this is [inaudible]. I took one of them and the smell was atrocious, so I threw it away, and I wasn't going to eat it. So the only thing I had a problem with was the fish dinner.*
Compliments	
Ease/convenience	*I'm alone, so that's it. So the answer would be it was helpful to me. It was very convenient. I have a microwave. If I just rip it open and put it in the microwave, then it was convenient. I didn't have to cook or do anything. So in that respect, it was very easy. And like I said, I'm alone. So it helped just me and there was no one else in there.*

Good experience	*And the only feedback I have about the food and what is kind of the obvious care that gets put into it, I really appreciate it…. No, but I'm going to say thank you for the lunches. It helps on so many levels*
	Yes. Yeah. It made me feel connected in the sense that I thought about the fact that it provided jobs to people in the kitchen to cook it, to cook the meals. And I thought about how the people in the kitchen, whoever was prepared, had to cook the meals, and they probably get paid and have a job. And then some people have to pack it up and so forth like that. So I felt as though some of the brochures or literature had pictures of people on it. So I felt like it was-- even though I didn't see them, I felt as though a little bit connected to them that I was on the receiving end of their hard work.

eTable 2: Themes and Illustrative Quotes Relating to the 'Dose' Dimension

Theme	Illustrative Quote
Feelings about meals for the household	*No, they wouldn't have eaten it… And they're very particular. My son is all grass-fed, all natural. And my daughter, I don't know. She's a flight attendant so she's never around, so…. Yeah. Pickiness and, yeah, whatever. Me, I'm not all that picky.*
	I think it would have made it easier for deciding what to do as a group for meals sometimes. So that way we didn't feel like we were kind of trying to make a split decision on who was getting what. Yeah, that would be kind of the only thing I would say about that part.
Challenges with food options	*I would think mostly is not the amount. We ate whatever was on the tray. But like I said, I was like, "Oh, gee, it has mushroom, but I do like what's in it, but it has mushroom." And I'm like, "Oh, I can't eat it." So it's a disappointment because you want to eat it, but you can't because-- yeah, that was a thing. That's where it comes for having a list of things that that would help a lot more….Yeah. Yeah. And no, pretty much everything was okay. But I would think my son, it was just like he had celiac. So things that he was a little bit to-- so his stomach couldn't tolerate as much. So I decided, "Well, it's all your luck. I'm going to keep mine."*
Reduced household labor	*I actually liked that idea. It made it a little bit easier as far as if I'm eating it, it's not. But I still have to make something else for somebody else in the house that we could all have a pre-made meal. So it wasn't like I had to do extra stuff.*
Helpful/easier for family	*I feel really good and very fortunate and lucky that I was able to qualify for this program. [When asked about household feelings] It was as helpful to him as it was to me….Yes. 100%. Thrilled. No*

	complaints.

eTable 3: Themes and Illustrative Quotes Relating to the "Delivery Method" Dimension

Theme	Illustrative Quote
Impact on Loneliness/Mental Health/Social Connection	
No impact on feelings of connection or social contact	*[when asked if participant experienced increase in social contact or connection] No....I didn't really interact with anyone. I just got the meals delivered and ate them.*
Increased feelings of connection or social contact	*[when asked how receiving meals impacted how connected the participant felt to others] Yeah, I do because it's somebody that is not coming because they have a problem with something. It's nice to just have somebody even just passing by and say, "Hi, hi." It picks up most of your days.*
Decreased loneliness	*[when asked if receiving meals made them feel less lonely] Absolutely. Yes…Because when you're sick, you feel like you are dependent. My situation is I've been sick for a couple of years and my stomach. And I feel isolated because of what I have to eat. And especially maybe in a week now, it's like they have to do stuff for you. Whereas with the food, it was more like "Hey, I'm going to have this and I just heat it up." So to me, it took me not always calling them or asking for help to make me something. I already had that was paper for me.*
No Impact on loneliness	*[Did receiving the meals ever make you feel less lonely] I wouldn't say so. I didn't really feel lonely, to be honest, so I don't really think that the meals kind of impacted that, I would say....Not necessarily. Just kind of felt the same throughout the process, I would say.*
Didn't interact much with delivery driver	*Well, it was a different person every week, pretty much, you know what I mean? So.*
Delivery driver was nice or considerate	*They were nice. If I was home, and I opened the door, "So could you put it inside the door because the boxes are heavy for this old bird?" I love those ice packs, too, I have to say.*
Delivery/shipping issues	*It was pretty good. The only thing I would say is my meals were delivered via UPS. And with the weather, sometimes it was left outside,*

	and I didn't know that they had came. So sometimes, it may have been out there for several hours. So some of the stuff had started-- the ice started melting, or if it was frozen, it was kind of melting.

eTable 4: Themes and Illustrative Quotes Relating to the Impact of the Intervention on Health and Economic Well-Being

Theme	Illustrative Quote
Differences to Health	
Improved dietary habits	*Yes, it has. I'm eating healthier than I was before, fast food, pizzas, things like that. I [inaudible] having cooked and not having the right ingredients inside of it, like less salt, things like that. So it definitely improves my health.*
Improved Hemoglobin A1c/blood sugar levels	*Well, I can tell you that when I first started the meal plan-- before the research, I actually got, I think, six months worth of meals through my dietician, filled about an application, and got food. So it's actually 190 pounds. I'm 4'10". So that was kind of overweight, diabetic, COPD. And because I had started the meal plan and my A1C was-- did I say it was 9.8? Yeah. My A1C was 9.8. So when I since I did the meal program, my A1C went all the way down to 5.7. I'm actually down 144 pounds. Yeah. So it made a huge improvement, huge difference in my life. Just eating the right way.*
	So it's actually 190 pounds. I'm 4'10". So that was kind of overweight, diabetic, COPD. And because I had started the meal plan and my A1C was-- did I say it was 9.8? Yeah. My A1C was 9.8. So when I since I did the meal program, my A1C went all the way down to 5.7. I'm actually down 144 pounds. Yeah. So it made a huge improvement, huge difference in my life. Just eating the right way.
Improved blood pressure	*...and my blood pressure has improved.*
Weight loss	*Well, it helped me regulate my weight so that I wouldn't eat a lot in between meals and eat things that weren't that good for me.*
Positive emotional and psychological impact	*I feel grateful that I have something to eat when I'm in pain. I feel that my health has improved because of these meals. I didn't eat before because I couldn't stand in front of a store or I couldn't go to the store to buy groceries because buying groceries is also a thing when you are in pain. You have to bring vegetables. You have to pick them up. You have to clean them. You have to cook. It's a*

	hassle when you are not able to. And this program has given me the proper nutrition that I deserve.
Non-Health Related Benefits	
Financial relief	*Aside from health, I mean, like I mentioned, it was helpful financially just knowing that we were able to have enough meals throughout the week that we could afford everything else still.*
Ease of accessibility and convenience	*And with the way I qualified for the program, it allows my husband to have meals as well. And I couldn't cook prior to this because I couldn't stand anymore. So it's saving us.*
Relief from food insecurity	*I think it was more that I had food and I didn't have to think of, "Okay. Well, you know I haven't been to the store." I have to wait until I go to the store to be able to eat something besides, say, peanut butter and jelly sandwich… Or just peanut butter and jelly if I'm out of bread.*
	With food stamps, only get maybe a week or two worth of food, and the rest of the month they have nothing. I lived out a lot. And when I got these meals, I didn't have to worry.
Helps general finances	*Well, I mean, one thing that I don't have to spend as much money on food each week. And that definitely helps.*
